# Global Initiative for Chronic Obstructive Lung Disease: The Changes Made

**DOI:** 10.7759/cureus.4985

**Published:** 2019-06-24

**Authors:** Avani R Patel, Amar R Patel, Shivank Singh, Shantanu Singh, Imran Khawaja

**Affiliations:** 1 Internal Medicine, Northern California Kaiser Permanente, Fremont, USA; 2 Internal Medicine, Southern Medical University, Guangzhou, CHN; 3 Pulmonary Medicine, Marshall University School of Medicine, Huntington, USA

**Keywords:** spirometry, smoking, abcd assessment tool, long-acting beta-2 agonist, global initiative for chronic obstructive lung disease, chronic obstructive pulmonary disease (copd), persistent airflow limitation, forced vital capacity, forced expiratory volume in one second, copd exacerbation

## Abstract

Chronic obstructive pulmonary disease or COPD is one of the conditions that physicians frequently see in both the hospital and outpatient setting. In order to improve diagnostic and treatment outcomes, the Global Strategy for the Diagnosis, Management and Prevention of COPD, the Global Initiative for Chronic Obstructive Lung Disease (GOLD) was created in 2001. Every year, a new report is generated based on an analysis of published studies which attempts to improve the way physicians handle COPD. GOLD reports are considered to be essential evidence-based reference tools for the implementation of effective management plans, and represent the current best practices for the care of patients with COPD. The 2017 report greatly revised the guidelines and added a few components that changed the system of COPD diagnosis and treatment. This review article addresses those changes, explains the current guidelines, and draws attention to areas that still require improvement.

## Introduction and background

**Figure 1:**
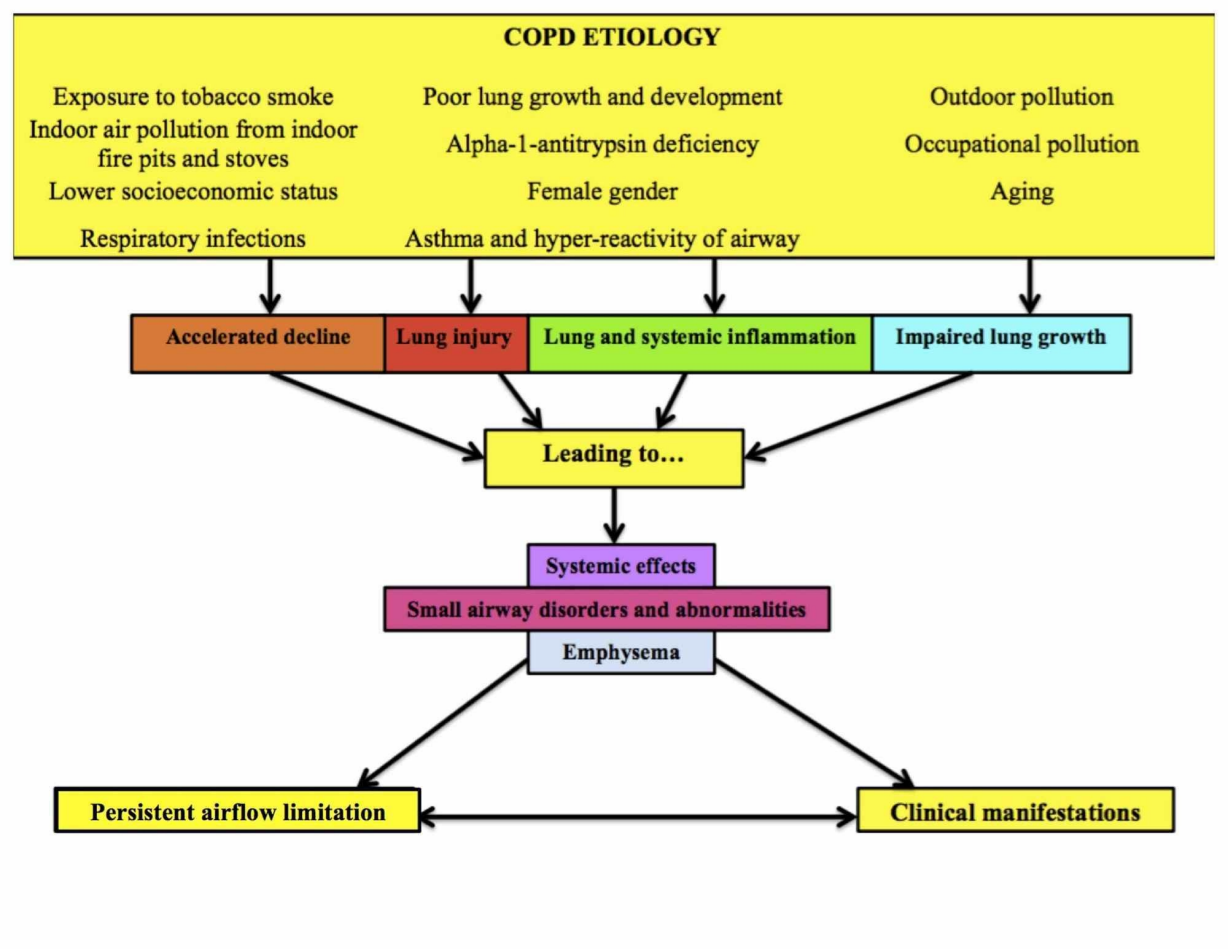
The Development of COPD This figure shows the development of COPD. The factors shown contribute to the development of airflow limitation and eventual to clinical manifestations of COPD [2]. COPD: chronic obstructive pulmonary disease.

Chronic obstructive pulmonary disease (COPD) has become a major concern for global health. COPD is defined as a preventable and treatable disease characterized by persistent airflow limitation that is usually progressive and associated with an enhanced chronic inflammatory response in the airways and the lung to noxious particles or gases [1]. This persistent airflow limitation leads to the development of COPD (see Figure 1) [2]. The airflow limitation is caused by a combination of both small airway disease (obstructive bronchiolitis) and parenchymal destruction (emphysema) [2]. Many patients will experience exacerbations or have comorbidities, which will lead to a worsening of their overall condition [3]. This worsening may be due to continuous exposure of the patient to known COPD risk factors like tobacco smoke, or a patient's non-adherence to the prescribed medical treatment. Patients with COPD will present with symptoms like dyspnea, chronic cough with or without sputum production, and possibly with a history of exposure to risk factors for the disease [2].

The risk of developing COPD increases with certain factors. These are exposure to tobacco smoke, usually through cigarettes, cigars, or other types of smoking. People living in poorly ventilated houses are exposed to large amounts of indoor air pollution from indoor fire pits and stoves used for cooking and heating [2]. This can also be indirectly linked to a lower socioeconomic status, respiratory infections, and poor lung growth and development [4-5]. Another risk factor is asthma and hyper-reactivity of airways, which can lead to repetitive airflow limitation [2]. Genetic factors may also contribute to COPD incidence, especially alpha-1-antitrypsin (AAT) deficiency [6]. The function of AAT is to protect the lungs from proteolytic damage by neutrophil elastase [2, 4]. Other risk factors include outdoor and occupational pollution, aging, and the female gender [2].

Previous studies have reported that COPD is the third-leading cause of mortality worldwide [7]. In the United States, COPD affects more than 15 million Americans and more than 140,000 people die each year from this disease [8]. Despite its prevalence, COPD is greatly underdiagnosed in the primary care setting [9].

This is where the Global Strategy for the Diagnosis, Management and Prevention of COPD, Global Initiative for Chronic Obstructive Lung Disease (GOLD), is imperative for physicians. Since 2001, GOLD reports have been utilized as evidence-based reference tools for the implementation of effective COPD disease management plans, and represent the current best practices for the care of patients living with the disease. This review article details regarding the major guideline changes seen in the 2017 report that changed the way COPD is treated. The new guidelines are very different from the old ones because they focus on symptom severity and exacerbation risk for COPD classification rather than the spirometry test findings, although spirometry is still essential for airflow limitation assessment. It has also several changes to the treatment algorithm.

## Review

Assessment of a COPD patient

For physicians, the goals of COPD assessment are to determine the disease severity, the degree of airflow limitation, and the decompensation risk [2]. Once these parameters are accurately deduced, it becomes easier to guide therapy. COPD patients may need management of comorbid conditions like cardiovascular disease, skeletal muscle dysfunction, metabolic syndrome, osteoporosis, depression, anxiety, and lung cancer [2]. In order to maintain optimal overall health for COPD patients, these comorbid conditions should be checked for and treated appropriately.

Physicians are required to take a thorough history and perform a comprehensive examination when patients are suspected to have COPD. Then the patient should undergo a spirometry test to confirm the presence of persistent airflow limitation [2]. Patients with FEV1/FVC (forced expiratory volume in the first second/forced vital capacity) ratio of less than 0.7 are considered to have airway obstruction [2]. Testing is also done after the administration of short-acting bronchodilator and needs a minimum of three spirometry measurements to minimize variability. [2]. Based on the measured values, the patient is then classified into one of four categories: GOLD 1, GOLD 2, GOLD 3, and GOLD 4. According to the 2017 revised GOLD guidelines, spirometry is no longer considered to have a strong correlation with the symptoms and impairment of the patient’s health status [10-11]. Because of this, a formal symptom assessment needs to be done [2]. 

The symptoms need to be assessed in order to classify their respective levels of severity. According the 2017 GOLD report, the COPD Assessment Test (CAT) and the COPD Control Questionnaire (CCQ) are used for this purpose [2]. These questionnaires assess each patient’s symptoms using a Likert scale. In previous years, the Modified British Medical Research Council questionnaire was recommended, and it is still used today by physicians despite not being the recommended questionnaire by the 2017 GOLD report [12].

The next important step of COPD assessment is analyzing the patient’s exacerbation history. Based on this, patients can be classified as high risk of exacerbation or low risk of exacerbation. Low risk of exacerbation is defined as one or less exacerbation not resulting in hospital admission in the previous 12 months while high risk of exacerbation is defined as a minimum of two exacerbations or any exacerbations resulting in hospital admission in the last 12 months [12].

**Figure 2:**
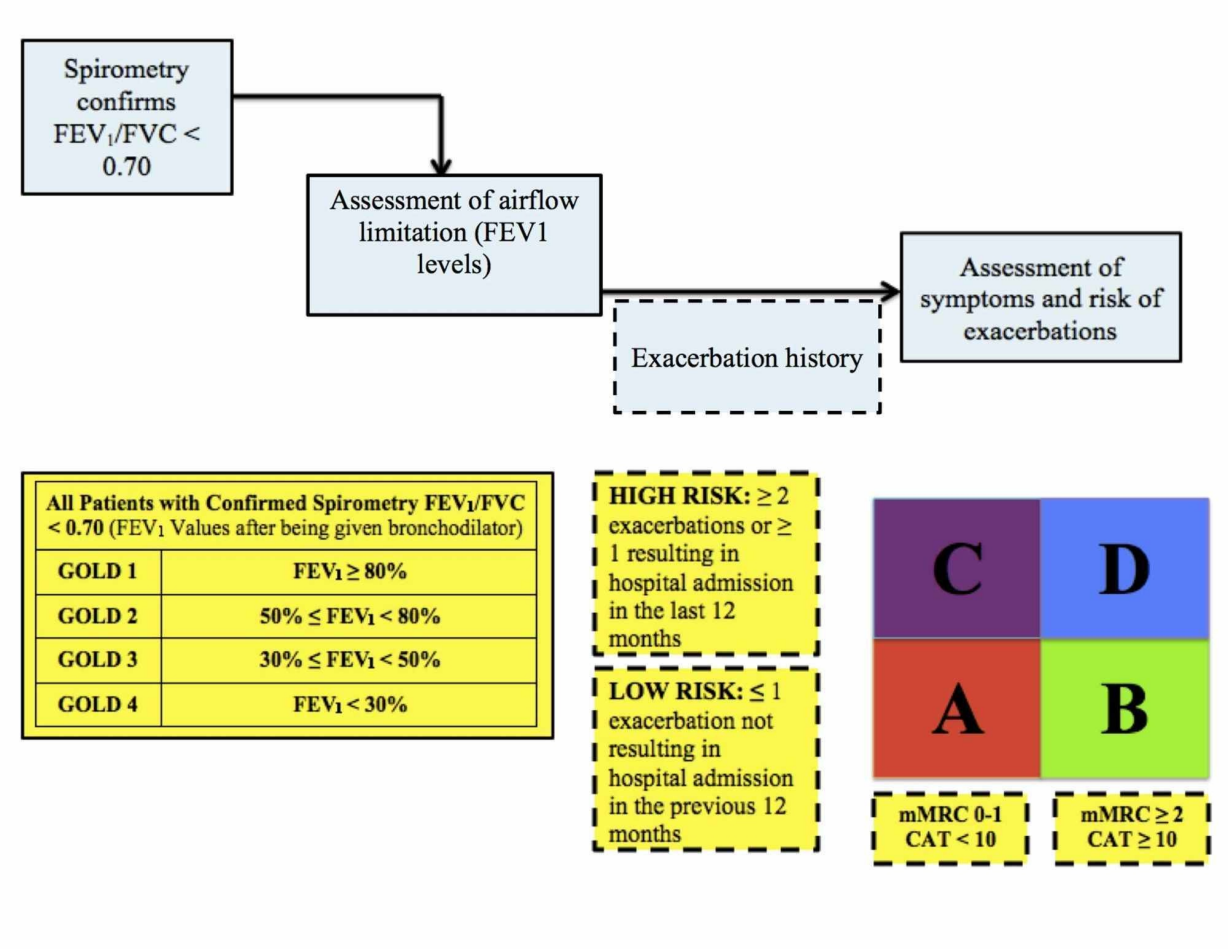
The Refined ABCD Assessment Tool This figure is representative of the refined ABCD assessment tool according to GOLD report 2017 along with the other important steps needed for patient classification [2].

Finally, the last step is to plug in the above-determined values in the refined ABCD assessment tool and determine the final classification of the patient (see Figure 2) [2]. Based on the patient’s FEV1 post bronchodilator result, symptom assessment score, and exacerbation history, patients can be classified accordingly in certain categories (see Table 1) [2, 12].

**Table 1:** COPD Classification According to Symptom and Severity Risk [2, 12] After using the refined ABCD assessment tool, patients are classified into one of the four above categories. This impacts their further management [2, 12].

Classification According to Symptom and Severity Risk
GOLD group A: low symptom severity, low exacerbation risk
GOLD group B: high symptom severity, low exacerbation risk
GOLD group C: low symptom severity, high exacerbation risk
GOLD group D: high symptom severity, high exacerbation risk

Treatment based on current GOLD guidelines

The goals of COPD management are to reduce severity and prevent exacerbation [2]. Based on the patient’s classification, treatment will be initiated. For patients in whom symptoms persist or exacerbations continue to occur, there are escalation strategies available.

Group A patients have low symptom severity and low exacerbation risk. They should be started with a single bronchodilator (beta-2 agonist), which will be either a long-acting beta-2 agonist (LABA) or short-acting beta-2 agonist (SABA) [12]. This should be escalated to an alternative class of bronchodilators (anticholinergics) if necessary for management [2, 12]. Patients should be made aware that beta-2 agonist drugs can precipitate cardiac rhythm disturbances and tremors in susceptible patients [2].

Group B patients have high symptom severity and low exacerbation risk. They should be started with a LABA or a long-acting antimuscarinic antagonist (LAMA) for monotherapy initially [2]. If the symptoms persist, then the treatment should be escalated to dual therapy with LABA and LAMA [12].

Group C patients have low symptom severity and high exacerbation risk. They need to be initially treated with a LAMA [2]. If exacerbations persist then the treatment is escalated to dual therapy with LAMA and LABA [12]. A second escalating option, although not preferred, is putting the patient on a combination of LABA and inhaled corticosteroids (ICS) [12].

Group D patients have both high symptom severity and high exacerbation risk. They should be started with LABA and LAMA or with LAMA monotherapy [12]. If symptoms or exacerbations persist then treatment should be escalated to LABA with ICS, and if that does not manage the patient, then LABA, LAMA, and ICS should be started as a triple therapy approach [12]. If the patient continues to have further exacerbations even on triple therapy, then roflumilast with or without a macrolide is given [12].

COPD patients are advised that smoking cessation remains important during their treatment, although the effectiveness and safety of e-cigarettes is uncertain at present [2, 12]. The inhaler technique of patients needs to be regularly assessed [2]. Patients with severe chronic hypoxemia at rest may need to be placed on long-term oxygen therapy [2]. Patients should ideally be vaccinated against pneumococcal bacteria and influenza [2].

The changes made

GOLD report 2017 has markedly revised the guidelines for COPD classification. Certain changes were made, which include basing the classification of COPD severity on clinical criteria alone and changing treatment options. Currently, GOLD recommends basing the COPD grade on the symptom assessment and the risk for exacerbation [2]. The ABCD tool has been refined, and CCQ and CAT questionnaires have now been added for symptom assessment. The report also includes changes in treatment strategy. 

New options for treatment include a contemporary set of escalation strategies for patients still having persistent symptoms or exacerbations. There are strong recommendations to use a combination of SABA with short-acting antimuscarinic antagonists (SAMA) rather than monotherapy [12]. Group A patients should be started on either LABA or SABA [2]. Group B patients should be started on LAMA monotherapy [2]. When LABA monotherapy is unsuccessful, then the 2017 report recommends that LABA and LAMA treatment should be started, rather than LABA and ICS [2].

Studies have been done to compare and contrast the new guidelines' effectiveness [13-15]. One objective has been to predict which patients will have exacerbations in the following year and compare that to previous guideline predictions [13]. It has been determined that there is a consistency in its ability to predict exacerbations in both current and previous guidelines [13]. The 2017 classification guidelines have caused a shift of COPD patients from category D to B or A [13-15]. Despite this, there is still a great deal of uncertainty regarding the new guidelines' impact on patient mortality and morbidity. More studies need to be done.

## Conclusions

This review article discusses the revision of COPD guidelines as per the Global Strategy for the Diagnosis, Management and Prevention of COPD, GOLD 2017 report. The 2017 guidelines no longer recommend spirometry as the sole diagnostic test for COPD diagnosis because even with its good sensitivity, it has a weak specificity. Therefore, symptom assessment and exacerbation history have changed how patients are diagnosed. Although there is inadequate data, it is perceived that a reclassification has been executed, all the potential variables have not been identified, and it is still unclear about whether patients are directly benefiting from the changed treatment according to their new classification. COPD is still a leading cause of mortality in the world and is greatly under-diagnosed and inappropriately treated. More studies are required, and as physicians it becomes important for us to understand the current stance on COPD classification and treatment. By learning this, we can address the deficiencies of the current classification and aid in its improvement.
